# Biomarkers in Colorectal Cancer: Clinically Relevant Diagnostic and Prognostic Molecular Features, and the Future of Precision Medicine

**DOI:** 10.3390/jpm16030132

**Published:** 2026-02-28

**Authors:** Rebecca Whitmer, Julia Sepulveda, Jason Gandhi, Isha Puri, Rohan Gupta

**Affiliations:** 1 Harris Methodist Hospital, Fort Worth, TX 76104, USA; juliasepulveda@texashealth.org (J.S.); ishapuri@texashealth.org (I.P.); 2 The Center for Cancer & Blood Disorders, Fort Worth, TX 76104, USA; rgupta@txcc.com

**Keywords:** colorectal cancer, biomarkers, personalized medicine, precision oncology, molecular profiling, diagnostic biomarkers, prognostic biomarkers, predictive biomarkers, tumor genomics, microsatellite instability, mismatch repair, circulating tumor DNA, targeted therapy, treatment stratification, translational research

## Abstract

Colorectal cancer (CRC) is a major public health concern in the United States. It is currently the fourth most diagnosed cancer and, despite advancements in screening and treatment, the second leading cause of cancer-related deaths. Approximately 153,000 new cases are diagnosed annually, with over 53,000 deaths reported. Understanding the molecular and genetic underpinnings of CRC biomarkers plays a crucial role in diagnosis, prognosis, and treatment planning. Specific gene mutations, including MMR deficiency leading to high microsatellite instability (MSI), as well as several other common mutations in CRC, including APC, TP53, KRAS, NRAS, SMAD4, PIK3CA and BRAF, provide valuable insights into tumor biology, therapeutic resistance, and response to targeted therapies. This review explores the mutations and co-mutations most relevant to CRC, their prevalence, prognostic significance, and implications for precision oncology. By focusing on these genetic and epigenetic alterations, we aim to contextualize how biomarker-driven strategies are reshaping the management of CRC in both early and advanced disease settings.

## 1. Introduction to Precision Medicine

Traditionally, treatment for CRC relied on histopathology and anatomical staging of the tumor. Treatment regimens consisted of nonselective cytotoxic chemotherapy agents, often empirically combined, and were given based on stage but not tumor biology, an approach which limited the ability to predict individual patient response to treatment and toxicity. In the early 2000s, a paradigm shift began to emerge with the development of precision oncology, which is the integration of molecular and genomic profiling to match therapies to the specific biomarkers of an individual patient’s tumor.

Precision oncology began to gain traction in the clinical realm shortly thereafter as molecular profiling demonstrated stratification of patients based on actionable biomarkers to select targeted therapies and immunotherapies, thereby reducing toxic exposure to ineffective treatments and improving overall survival [1]. Later, it would become the standard of care through continued advances in next-generation sequencing and the integration of molecular diagnostics into treatment algorithms as recommended by major societies such as the National Comprehensive Cancer Network (NCCN), which recommends molecular profiling for all patients diagnosed with metastatic CRC, to individualize treatment selection [2].

The molecular landscape of CRC remains vast and complex with seemingly endless variability in the molecular heterogeneity of tumors found within one individual, let alone the almost 150 k novel cases identified annually. Through precision oncology, advancements have been made which allow researchers to understand some of the disruption occurring at various signaling pathways and identify established and emerging biomarkers which could further help to refine diagnosis, improve treatments and, most importantly, improve survivability [1].

## 2. DNA Mismatch Repair Gene Mutations

During DNA replication, genes called mismatch repair (MMR) genes help maintain the integrity of copied DNA material by removing mismatched base pairs that occur sporadically during normal DNA replication. The human genome contains many of these MMR genes to accurately replicate DNA, but the most common MMR genes to be implicated in CRC are MLH1, MSH2, MSH6, and PMS2 [3]. MMR genes can undergo either germline or sporadic mutation, which leads to inaccurate DNA replication and an increased risk of developing CRC. About 15% of all CRCs have MMR gene mutations—of these 15%, about 12–13% are sporadic (or acquired) mutations. The remaining 2–3% are considered germline (or inherited) mutations associated with an inherited CRC called hereditary non-polyposis CRC (HNPCRC) [4].

HNPCRC is rare and accounts for only 3% of all CRCs. Generally, HNPCRC is diagnosed via a combination of family history, immunohistochemical (IHI) staining, and analysis of the tumor tissue for microsatellite instability [4]. IHI staining in HNPCRC typically shows low MMR activity due to deficient MMR gene function and high microsatellite instability (MSI). Regardless of family history, many institutions are now universally testing for MMR deficiencies and high MSI, an approach that is both important for treatment planning as well as for identifying patients who need to undergo further evaluation for HNPCRC.

Sporadic (acquired) MMR gene mutations are far more common than germline MMR mutations. The most common mechanism by which these sporadic MMR gene mutations occur is via hypermethylation of the MLH1 promotor. This hypermethylation effectively turns off production of the MLH1 protein, which inhibits appropriate MMR function, leading to hypermutability and high microsatellite instability [5].

## 3. Microsatellite Instability

When DNA does not have functioning MMR genes, the DNA is able to replicate without repair and becomes hypermutable. This is referred to as high microsatellite instability (MSI). Tumors that are characterized by high MSI are most commonly found in the proximal colon and carry a slightly better prognosis than tumors with low MSI. Tumors with high MSI are so hypermutable that they are more easily recognized by both the body’s immune system, and by immunotherapies, as ‘non-self’, often making them more responsive to treatment and thus improving prognosis. This is why microsatellite stable tumors often carry a worse prognosis [6].

Many chemotherapies work by acting on certain target genes. High MSI causes hypermutable tumors that can inactivate these target genes, which modifies a tumor’s sensitivity to certain immunotherapies. Multiple studies have supported the idea that MSI-driven inactivation of target genes can modify tumor chemosensitivity [7,8]. Further, many studies have suggested that tumors with high MSI have improved prognosis and are less likely to metastasize [9,10].

## 4. Common Mutations in CRC

CRC occurs via the gradual accumulation of gene mutations that cause either the deactivation of tumor suppressor genes or the activation of oncogenes. In this chapter we provide an overview of some of the most common mutations seen in CRC: APC, TP53, KRAS, NRAS, SMAD4, PIK3CA and BRAF.

Briefly, mutations in APC and TP53, both powerful tumor suppressors, seem to be encountered earlier on in tumorigenesis and are seen in a majority of adenomas and carcinomas. Mutations in KRAS—a gene involved in cell division regulation—also occur frequently, in about 30–50% of CRC cases, and are important for prognostic and therapeutic decision making. NRAS, another protein involved in the regulation of cell division, mutates much less frequently but is also associated with poorer prognosis and metastasis. SMAD4, a tumor suppressor gene, is also similarly associated with poor prognosis and metastasis. Lastly, both PICK3CA and BRAF mutations are also less commonly encountered and are mostly seen in right-sided tumors. BRAF mutations are associated with high MSI tumors [11].

Some of these genetic markers can help predict overall survival, response to treatment, and most importantly, help direct treatment plans [3,12]. There are nuances associated with these various mutations and genetic markers, which are summarized in Table 1 below.

## 5. APC Mutation

Adenomatous polyposis coli (APC) is a tumor suppressor gene which is key in regulating colonic epithelial cell proliferation, differentiation and apoptosis accomplished through regulation of the Wnt/β-catenin signaling pathway. APC also has roles within the cell responsible for cell migration and chromosomal stability.

The American College of Medical Genetics and Genomics describes the APC gene as a ‘Gatekeeper’ gene given loss of function mutations resulting in dysregulation of the Wnt/β-catenin signaling pathway; promoting the growth of adenomas is the first event in the ‘adenoma-carcinoma’ sequence. This sequence is a series of events that leads to dysregulated cell proliferation, chromosomal instability, and accumulated mutations, ultimately causing high-grade dysplasia and transformation into carcinomas [13].

### 5.1. Frequency and Prognosis

APC mutations are found in 80–85% of sporadic CRC and almost universally in familial adenomatous polyposis (FAP). The prognostic implications of APC mutations are highly context-dependent [14]. For example, in a study by Jorissen et al., in microsatellite stable (MSS) proximal colon cancers, the presence of APC mutated tumors was associated with increased overall survival, compared to tumors with wild-type APC [15]. In contrast, a study by Osumi et al. showed the presence of APC mutations in advanced-stage CRC, and metastatic CRC was associated with a worse prognosis [16].

Whether the mutation is somatic or germline can also affect possible prognosis given the difference in pathogenesis of the disease. Hereditary syndromes like FAP carry an almost 100% risk of transformation to CRC if untreated, whereas in somatic disease, additional mutations are needed for the progression to carcinoma to occur, in a phenomenon known as the two-hit hypothesis [14].

### 5.2. Targeted and Non-Targeted Therapy

Standard management for patients with FAP or attenuated FAP includes lifelong endoscopic surveillance and polypectomy as recommended by the American Society for Gastrointestinal Endoscopy. When extensive polyposis is documented or there is a high concern for cancer, prophylactic colectomy or proctocolectomy is recommended. Targeted therapies for APC mutations are not yet a standard of care, though current research has shown APC mutation status may be a positive predictor for EGFR inhibitor response in CRC, particularly when present in conjunction with TP53c mutations [17].

## 6. TP53 Mutation

Normally, the TP53 gene encodes the tumor suppressor protein TP53 which is responsible for regulating cell cycle arrest, apoptosis, as well as DNA repair in response to stressors. TP53 gene mutations are present in approximately 50–60% of all CRC with four main types of mutations seen including missense, nonsense, frameshift and splice site mutations.

Missense mutations are the most common at ‘hotspot’ codons 175, 248 and 273. Missense mutations can cause both loss function and/or gain of function mutations, such as cell proliferation and even metastasis. Because TP53 is typically a critical tumor suppressor, its inactivation is typically a driver of malignant transformation in CRC and is associated with more advanced disease, poor prognostic features including vascular or lymphatic invasion, and altered or poor response to therapy [17].

### 6.1. Frequency and Prognosis

Although there is potentially a correlation between germline TP53 mutations and increased rates of early-onset CRC which has been shown in some studies and databases, there are no current recommendations to utilize TP53 mutations as a prognostic marker for CRC. However, certain hotspot mutations (e.g., R273) appear to be correlated with increased metastatic potential and poor outcomes when compared to other TP53 mutations [18].

### 6.2. Potential Treatment Strategies

Although there are currently no approved therapies that treat cancers associated with TP53 mutations or deletions, there are several ongoing clinical trials that demonstrate the efficacy of potential new therapies, particularly the phase II FOCUS-4C trial, which evaluates the efficacy of adavosertib, an inhibitor of cell cycle regulator Wee1, which has shown potential for increasing progression-free survival in stable post-chemotherapy CRC tumors with TP53-KRAS co-mutations [19].

## 7. RAS Mutations

Primarily encoded by three highly homologous isoforms, KRAS, NRAS and HRAS, collectively known as the ‘RAS proteins’ are proto-oncogenes responsible for signaling cell survival, division and proliferation. The proteins in this signaling network are tightly regulated GTPases cycling between active and inactive confirmations facilitated by guanine nucleotide exchange factors (GEF) and GTPase-activating proteins (GAP), respectively. Worldwide, approximately 3.4 million newly diagnosed malignancies are attributed to RAS mutations [20,21].

Typically noted at codons 12, 13 and 61 with a 97% incidence, mutations in the RAS pathway do not change expression but rather functionality. These gain-of-function missense mutations produce constitutive activation of RAS and are widely observed in cancer, approximately 19% of human malignancies [20]. RAS mutations, particularly in the KRAS and NRAS genes, are found in about half of all CRCs.

## 8. KRAS Mutations

KRAS encodes a family of RAS proteins that function as GTPases. As stated above, these mutations, occurring typically in codons 12 and 13 of exon 2, lead to constitutive activation of the RAS/MAPK signaling pathway, promoting uninhibited tumor cell proliferation and survival [21].

### 8.1. Frequency and Prognosis

KRAS mutations occur in approximately 40% of CRCs and are more frequently observed in right-sided tumors. KRAS mutations are associated with worse prognosis and more limited therapeutic options, mostly due to their resistance to EGFR-targeted therapies. Tumors without the KRAS mutation (KRAS wild-type tumors), especially those located on the left side of the colon, respond more favorably to EGFR inhibitors and generally have better outcomes when treated appropriately [20,21,22].

### 8.2. Targeted Therapy

For KRAS wild-type metastatic CRCs, anti-EGFR monoclonal antibodies—cetuximab or panitumumab—offer significant therapeutic benefit, especially when combined with chemotherapy. These agents have demonstrated efficacy in improving progression-free and overall survival, particularly in left-sided primary tumors. On the other hand, patients with KRAS-mutant tumors are generally not candidates for anti-EGFR therapy but may benefit from other targeted agents as discussed below [23].

### 8.3. Non-Targeted Therapy

Chemotherapy remains a foundational component of CRC treatment regardless of KRAS mutation status. Standard regimens include FOLFOX (5-fluorouracil, leucovorin, oxaliplatin), FOLFIRI (5-fluorouracil, leucovorin, irinotecan), or CAPOX (capecitabine and oxaliplatin). These can be used alone or in combination with targeted therapies depending on molecular profile and disease characteristics. Bevacizumab, a VEGF inhibitor, is often used in both KRAS-mutant and wild-type CRC, as its mechanism is independent of EGFR pathway status [20,21,23].

### 8.4. Additional Information

There are emerging treatments for KRAS-mutant CRC, particularly for KRAS G12C mutations, with the advent of G12C inhibitors such as Sotorasib, which was recently approved as a second-line therapy for chemorefractory colorectal cancer, when given in conjunction with EGFR inhibitors like panitumumab. This combination has been shown to improve progression-free survival [24].

## 9. NRAS Mutations

### 9.1. Frequency and Prognosis

Though KRAS mutations are encountered in approximately 40% of CRCs, NRAS are observed in a much smaller percentage of about 3–5% and seem to develop at a later stage of malignancy. They are associated with a poor prognosis and an impaired response to anticancer therapies as generally all RAS mutations are [22,25].

### 9.2. Targeted and Non-Targeted Therapy

NRAS mutations are somatic or non-hereditary, with the most common mutations being G12C, G12D, Q61K, and Q61R. For all newly diagnosed CRCs, biomarker testing should be performed to narrow down appropriate treatment. For example, NRAS mutations cause constitutive activation of the MAPK pathway as previously described. Because the NRAS mutation occurs downstream of EGRF, tumors with NRAS mutations have resistance to EGFR inhibitors, making the treatment ineffective. The current standard treatment for NRAS-mutated CRC is combination chemotherapy with FOLFOX, CAPOX, and or FOLFIRI with or without the monoclonal antibody Bevacizumab, an anti-angiogenic agent which blocks VEGF and inhibits the formation of blood vessels by the tumor [26].

## 10. PIK3CA Mutation

PIK3CA encodes the p110α catalytic subunit of phosphatidylinositol-4,5-bisphosphate 3-kinase (PI3K), a key component of the PI3K/AKT/mTOR intracellular signaling pathway that regulates cellular growth, metabolism, and survival. Somatic PIK3CA mutations in CRC cluster primarily at two hotspot regions—exon 9 (helical domain, e.g., E542K, E545K) and exon 20 (kinase domain, e.g., H1047R, H1047L). These substitutions confer constitutive PI3K activation, leading to downstream phosphorylation of AKT and mTOR and ultimately promoting oncogenic proliferation, epithelial–mesenchymal transition, and resistance to apoptosis [27].

### 10.1. Frequency and Prognosis

PIK3CA (phosphatidylinositol-4,5-bisphosphate 3-kinase catalytic subunit alpha) is among the recurrently mutated oncogenes in CRC, with somatic mutations reported in roughly 15–20% of tumors. The prognostic impact of PIK3CA mutations in CRC has been heterogeneous across studies: while some cohorts show little independent effect on overall survival after adjustment for stage and co-mutations, other analyses link PIK3CA alterations to treatment resistance and worse outcomes in specific clinical contexts. Importantly, PIK3CA status has emerged not only as a potential prognostic marker but also as a predictive marker for response to non-oncology agents [28].

### 10.2. Targeted and Non-Targeted Therapy

Direct targeting of PIK3CA-mutant CRC with PI3K inhibitors has been explored in early-phase trials, and while PI3K inhibitors (for example, alpelisib in other tumor types) demonstrate target engagement, single-agent activity in unselected CRC has been limited; combination approaches (PI3K blockade plus MAPK/MEK pathway inhibitors, anti-EGFR regimens, or cytotoxins) remain under investigation [29].

A clinically actionable, non-oncologic intervention that has gained substantial attention is low-dose aspirin: observational data previously suggested improved survival among patients with PIK3CA-mutant CRC, and a recent randomized adjuvant trial reported a sizeable reduction in recurrence for patients whose tumors harbored PI3K pathway alterations treated with low-dose aspirin versus placebo. Taken together, routine clinical use of PI3K small-molecule inhibitors for CRC is not established, whereas PIK3CA testing can be considered to identify patients who may benefit from adjunctive aspirin in the adjuvant setting pending guideline incorporation and individual bleeding-risk assessment. Any consideration of aspirin for secondary prevention in PIK3CA-altered CRC must balance anti-tumor benefit with gastrointestinal and hemorrhagic risks and should be undertaken with multidisciplinary input [28].

### 10.3. Additional Information

PIK3CA mutations commonly co-occur with other driver alterations (for example, KRAS) and may modulate tumor biology through enhanced PI3K/AKT/mTOR signaling, influencing proliferation, metabolism, and therapeutic resistance. This co-mutation and its clinical relevance will be discussed in further detail below.

## 11. SMAD4 Mutation

SMAD4 (Mothers Against Decapentaplegic Homolog 4), located on chromosome 18q21, encodes a central mediator of canonical TGF-β signaling. In normal colonic epithelium, TGF-β activation phosphorylates SMAD2/3, which complexes with SMAD4 to translocate to the nucleus and induce transcriptional programs controlling growth inhibition and differentiation. SMAD4 loss-of-function mutations (nonsense, frameshift, or missense within the MH2 domain) or homozygous deletions abrogate this tumor-suppressive signaling, promoting epithelial proliferation, genomic instability, and metastasis.

### 11.1. Frequency and Prognosis

Somatic SMAD4 mutations occur in about 10% of CRCs, while SMAD4 deletions occur in about 40% of CRCs. Loss or mutation of SMAD4 is consistently associated with adverse clinicopathologic features—more advanced stage at presentation, higher risk of nodal and distant metastasis, and inferior disease-free and overall survival in multiple cohorts—making SMAD4 an adverse prognostic biomarker in CRC [30].

### 11.2. Targeted and Non-Targeted Therapy

There are presently no SMAD4-directed therapies in routine clinical practice. Therapeutic strategies for SMAD4-deficient tumors are focused on exploiting downstream vulnerabilities (for example, targeting pathways that become dysregulated with TGF-β/SMAD axis loss) or using combination regimens to overcome associated chemoresistance. Preclinical and early-phase clinical work has evaluated approaches such as synthetic-lethality screens, inhibitors of compensatory signaling pathways, and immune-modulatory strategies; however, none have produced approved, SMAD4-specific agents to date. Clinically, identification of SMAD4 loss can inform prognosis and clinical trial selection and may influence discussions about intensified surveillance or adjuvant therapy strategies in high-risk patients [30].

### 11.3. Additional Information

SMAD4 aberrations often arise later in CRC progression and can co-exist with other pathway alterations; mechanistically, SMAD4 loss disrupts TGF-β-mediated growth inhibition and promotes a more invasive phenotype, stromal remodeling, and altered tumor–microenvironment interactions. Loss of SMAD4 has also been implicated in resistance to certain cytotoxic and targeted therapies in preclinical models, and it may correlate with distinct metastatic patterns (for example, increased propensity for liver metastases in some datasets).

## 12. BRAF Mutation

BRAF gene mutations, particularly the V600E substitution at codon 600, are present in fewer than 10% of sporadic CRCs. BRAF encodes a serine/threonine kinase that plays a key role in the MAPK/ERK signaling pathway, which regulates cellular growth, proliferation, differentiation, migration, and survival [31].

### 12.1. Frequency and Prognosis

BRAF mutations are found in approximately 10% of sporadic CRC cases. They are more frequently observed in older patients, current or former smokers, and are associated with right-sided tumors [31]. The BRAF V600E mutation is a well-established adverse prognostic marker in CRC associated with poorer outcomes and reduced survival, particularly in patients with metastatic disease and microsatellite-stable tumors. In contrast, non-V600E BRAF mutations, such as D594G, do not appear to confer the same negative prognostic impact [32].

### 12.2. Targeted and Non-Targeted Therapy

Combinational therapies involving BRAF inhibitors, EGFR-targeting antibodies, and MEK inhibitors have shown significant clinical benefit in patients with BRAF V600-mutated metastatic CRC [33,34]. The clinical success of triple therapy is likely due to conferred BRAF inhibitor resistance, which a tumor quickly develops when given BRAF inhibitors alone. When a BRAF V600 inhibitor is used as monotherapy, it suppresses MAPK signaling temporarily, removing negative feedback loops and ultimately causing rapid feedback reactivation of EGFR, which ultimately leads to the bypassing of the BRAF blockade [34,35].

Thus, standard first-line treatment options for BRAF-mutated CRC include chemotherapy regimens such as FOLFOX (5-FU, leucovorin, and oxaliplatin), FOLFIRI (5-FU, leucovorin, and irinotecan), and capecitabine plus oxaliplatin, followed by BRAF inhibitors, such as encorafenib, and anti-EGFR antibodies such as cetuximab. Despite triple therapy, BRAF inhibitor resistance remains a clinical challenge [34]. BRAF V600E mutations do not confer resistance to anti-VEGF therapy; thus, patients with BRAF-mutated tumors appear to benefit from agents such as bevacizumab to a similar extent as those with BRAF wild-type tumors [36].

### 12.3. Additional Information

In some cases, the BRAF V600E mutation has been detected at the time of disease progression, suggesting a role in primary or acquired resistance, and is often more prevalent in tumors from non-responders to EGFR-targeting antibodies [37,38].

## 13. Co-Occurring Mutation Patterns

### 13.1. The Molecular Constelation of a Tumor

Above, we have discussed the most common mutations that occur in CRC; however, to fully understand the clinical relevance of tumor mutation patterns, it is critical to understand that individual tumor mutations do not occur in a vacuum. Indeed, these mutations often occur simultaneouly and interact, meaning that certain combinations—or constellations—of mutations can influence a tumor’s behavior, prognosis, therapeutic response, and mechanisms of resistance. Often, this is referred to as the ‘molecular constellation’ of a tumor. When mutations occur in tandem, they can amplify oncologic signaling and even induce resistance to certain therapies. Below, we will briefly discuss the most common co-mutations, including information on prevelance, clinical relevance, and treatment [39].

### 13.2. TP53 + KRAS Co-Mutations

Concurrent TP53 and KRAS mutations are associated with a more aggressive tumor biology, a worse overall survival, and more chromosomally unstable tumors. This is likely because KRAS mutations function as proliferation drivers, while TP53 mutations primarily inhibit cell apoptosis, meaning that in combination, tumors with both these mutations occuring simultaneously can both proliferate and avoid cell death [40].

### 13.3. BRAF V600E + High MSI Co-Mutations

BRAF V600E mutations commonly co-occur with sporadic high-MSI tumors caused by MLH1 promoter hypermethylation, as discussed above in Section 3. Due to rapid, unchecked proliferation, tumors with this co-mutation tend to posses a high tumor mutational burden, causing them to be easily recognized by the body’s own immune system [37]. Thus, tumors with this co-mutation are highly responsive to immunotherapy. Two landmark studies, KEYNOTE-144 [39] and Checkmate-142 [41], in particlar, offered insights into the significant benefits of immunotherapy in the setting of these co-mutations, contributing to the establishment of immunotherapies such as Pembrolizumab (Keynote) or nivolumab + ipilimumab (Checkmate 8HW) as first-line treatments in high-MSI metastatic CRC [42].

### 13.4. PIK3CA + KRAS Co-Mutations

PIK3CA and KRAS mutations are both oncogenes that promote unchecked cell growth and have a synergestic effect that enhances tumorgenesis and cell proliferation. In conjunction, they confer a worse prognosis [43,44], and currently, there are few targeted options. Tumors with this co-mutation could benefit from combination therapies with PI3K inhibitors and KRAS inhibitors, which are currently approved for refractory metastatic CRC [45,46].

### 13.5. Tumor Heterogeneity in CRC

Beyond co-mutations, it is also important to recognize the limitations of solid biopsies (tissue samples) in the setting of colorectal cancer, which is known for its heterogenous nature. This means that one area of a CRC tumor may posses a completely different mollecular constellation than another area on the same tumor. Indeed, this is only one serious limitation in solid biopsies, with another being the difficulty of surgical procurment of a tissue sample, and a third being that, due to the proliferative nature of CRCs, a tumor’s molecular biology is ever-changing. It is simple not feasible to collect multiple tissue samples at multiple intervals during the disease process. Instead, a process referred to as liquid biopsy is commonly used in CRC. Liquid biopsy is the process by which circulating tumor DNA is detected in the blood, and this process is used to aid in diagnosis, monitoring, and recurrence detection [47].

## 14. Conclusions and an Eye Toward the Future

Since the development of foundational anticancer agents such as 5-fluorouracil (5-FU) in the late 1950s, major advancements in the diagnosis and treatment of CRC have been achieved. Continued therapeutic advancements in the field of precision oncology have resulted in the development of effective molecular target drugs, thus increasing available treatment modalities and improving overall survivability by 24–36 months, even in patients with metastatic CRC.

With improved screening modalities and improved screening guidelines, CRC is now being detected in earlier stages; however, there has also been a notable increase in early-onset CRC (EOCRC) in the under-50 population. Epidemiologic data shows the increased incidence of EOCRC has risen independently of the aforementioned factors and was on the rise prior to these guideline changes being made, hence the rationale behind earlier screening [48]. While some cases are indeed being detected sooner due to updated screening guidelines, this alone does not fully explain the rising incidence of EOCRC.

Instead, EOCRC is likely secondary to a multitude of risk factors including genetics, lifestyle, dietary habits, environmental exposures, and underlying co-morbidities such as ulcerative colitis. Many key risk factors for EOCRC have been identified, including family history, smoking, and metabolic syndrome. Modifiable factors such as diet, lifestyle, alcohol consumption, smoking and obesity may offer potential for prevention in high-risk populations. These findings underscore the importance of identifying modifiable and non-modifiable risk factors to streamline early screening for individuals at higher risk for EOCRC [47,48].

While this review has been focused on a biomarker-driven understanding of CRC, future investigations into inflammation-associated CRC, as found in ulcerative colitis (UC), are warranted. The risk of CRC is significantly higher for patients with UC, with an increasing risk the longer a patient has UC. This is due to perpetual inflammation leading to high cell turnover, which ultimately leads to mutations and CRC. In UC-driven CRC, TP53 is notably an earlier mutation, whereas in sporadic CRC, TP53 mutations typically occur much later in the mutation cascade. In addition, while APC and KRAS mutations occur earlier in sporadic CRC, they often appear much later, or not at all, in UC-driven CRC [49].

Biomarker-driven treatment modalities have indeed reshaped diagnosis, prognosis, and treatment planning for patients affected by both EOCRC and CRC. Due to advancements in molecular profiling in CRC, now more than ever, patients have access to a larger variety of medications and treatment options with less toxic side effects and greater chances of survival and remission than in decades past. While truly individualized treatment throughout a patient’s entire disease course is not yet entirely feasible due to the complex heterogeneity of CRC, continued studies into more comprehensive molecular profiling and the shift towards precision medicine is already driving drug development and shifting the design and implementation of clinical trials. In the future, improved understanding of the dynamic changes involved in colorectal tumors at the molecular level at every disease stage will allow for improved treatment with molecular target drugs or immunotherapy.

## Figures and Tables

**Table 1:** Mutation summary including available targeted therapies and prognostic factors.

Mutation	Frequency	Targeted Therapy	Prognostic Information	Tumor Location
APC	~70–80%	None	Initiating event; not strongly prognostic	Left > right colon
TP53	~50–60%	None	Associated with tumor progression; poorer prognosis	Left > right colon
KRAS	~40–50%	VEGF Inhibitors	Worse prognosis	Both, slightly left-favored
NRAS	~2–5%	Resistance to EGFR inhibitors; no targeted therapy	Worse prognosis	Left colon
PIK3CA	~10–20%	PI3K inhibitors (under study)	Worse with KRAS co-mutation	Right > left colon
SMAD4	~10%	None	Worse prognosis; metastasis, poor differentiation	Left > right colon
BRAF (V600E)	~10%	BRAF + EGFR inhibitors	Poor prognosis, right-sided, often MSI-H	Right colon

## Data Availability

No new data were created or analyzed in this study.
